# Enhancing the speed of DNA walkers through soft confinement

**DOI:** 10.1038/s41598-025-93269-x

**Published:** 2025-03-19

**Authors:** Mathew O. Ogieva, Wolfgang G. Pfeifer, Sebastian Sensale

**Affiliations:** 1 Department of Physics, Cleveland State University, Cleveland, OH 44115 USA; 2 Department of Biochemistry and Molecular Biology, The University of Chicago, Chicago, IL 60637 USA; 3 Department of Mechanical and Aerospace Engineering, The Ohio State University, Columbus, OH 43210 USA; 4 Department of Physics, Indiana University Indianapolis, Indianapolis, IN 46202 USA

**Keywords:** DNA nanotechnology, Polymers

## Abstract

Over the past two decades, dynamic DNA origami structures have emerged as promising candidates for nanoscale signal and cargo transport. DNA walkers, programmable nanostructures that traverse tracks made of DNA, represent a key innovation in this field, enabling controlled and directional movement at the nanoscale. Despite relatively fast diffusion rates, the speed of DNA walkers remains constrained by the reaction-limited nature of strand exchange mechanisms, which depend both on the foothold-walker affinity and on the probability of the molecules being found close enough to bind. In this study, we explore how spatial confinement can expedite walker motion and evaluate two strategies to achieve this: the introduction of tailed DNA footholds, promoting pseudo-rotational dynamics, and the addition of walls along the DNA track, promoting pseudo-curvilinear dynamics. Using simulations and stochastic theories, we demonstrate that, by reducing the sampling of conformations far from the binding sites, tailed footholds provide the best speed enhancement, achieving a fourfold increase in speed. Trench-like confinement yields a more modest threefold increase, what, while significant, requires extensive structural modifications to the DNA track, limiting design flexibility and reducing cost-efficiency in comparison to the tailed footholds. The combination of tailed footholds and trench-like confinement turns the walker-foothold system bistable, with two distinct stable states separated by an energy barrier. By focusing on the properties of the DNA track, this study offers novel insights into leveraging soft structural motifs to optimize signal propagation rates, with implications for sensing, robotics and molecular computing in reaction-diffusion systems.

## Introduction

DNA nanotechnology has rapidly emerged as a versatile and advancing field with growing applications across various domains, including bio-sensing, nano-robotics, gene and drug delivery, and plasmonics ^1–7^. A significant milestone in this field is the transition from static to dynamic systems, enabling the development of devices capable of undergoing structural transformations in response to external stimuli ^8–10^. This advancement has opened pathways for fabricating synthetic devices that autonomously execute predefined tasks through controlled structural changes, representing a key goal in dynamic DNA nanotechnology ^11^.

**Fig. 1:**
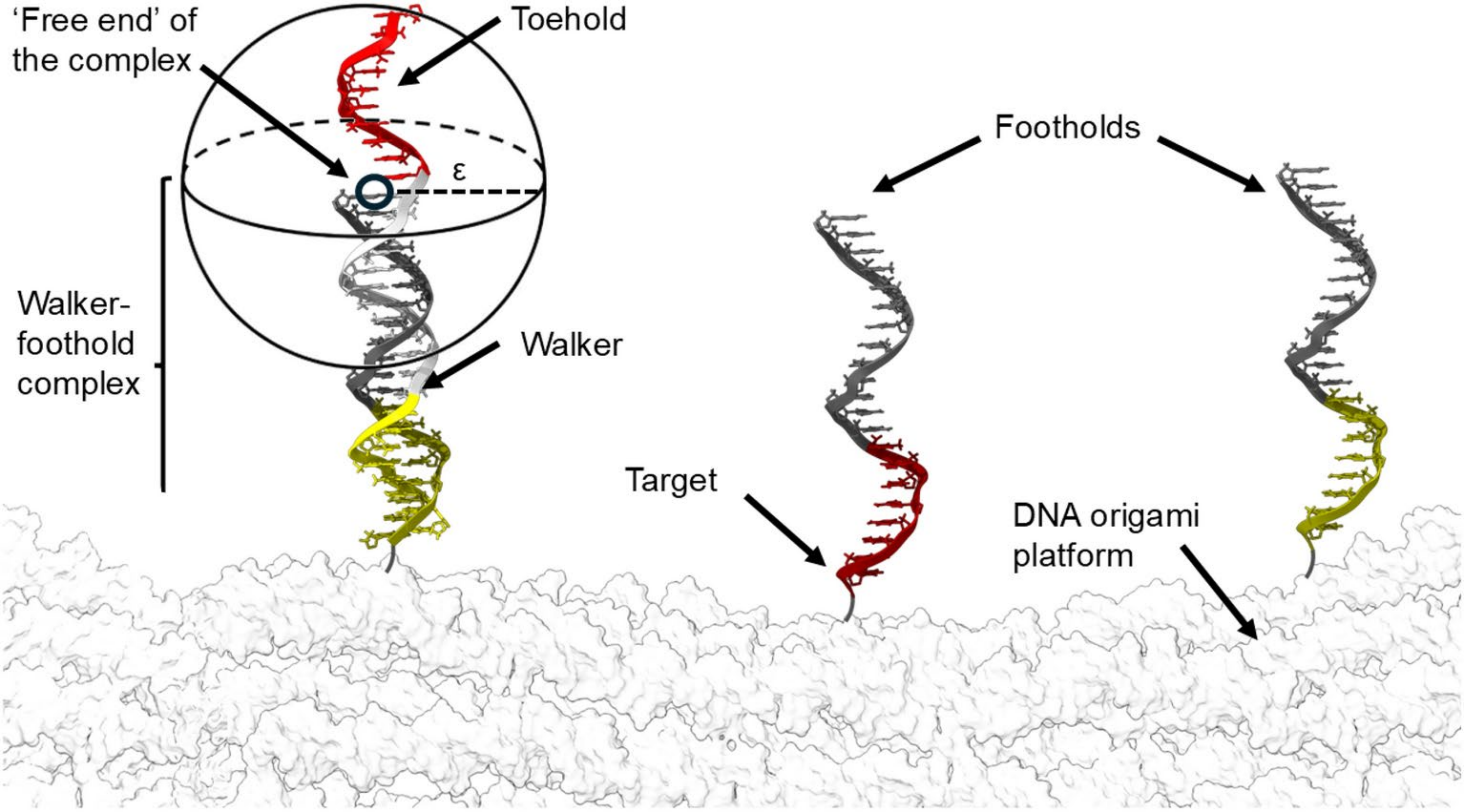
Schematic of the DNA walker system described by Li et al.^20^, including the nomenclature for elements referenced in this article. Red domains are engineered to bind specifically with red domains, gray domains with gray domains, and yellow domains with yellow domains.

One prominent example of such dynamic systems is the DNA walker, a nanomachine composed of multiple DNA domains that interacts with a DNA-based track through binding and unbinding reactions ^12,13^. Various DNA walker designs have been proposed and experimentally demonstrated ^12,14,15^, yet their operational speeds remain far below those of natural molecular motors, such as kinesin, myosin, and dynein ^16–19^. Notable progress was achieved by Li et al.^20^ with the introduction of a cartwheeling DNA walker, which is composed of distinct functional regions designed to facilitate autonomous movement across a surface of complementary single-stranded (ss) DNA sequences (‘footholds’) via toehold-mediated strand displacement ^21^. The substrate comprises two distinct types of complementary footholds, with the walkers alternating between binding to one type and the other (see Fig. 1). This alternation drives the cartwheeling motion, enabling the walkers to move ‘head-over-heels’ between binding sites. The length of the walkers, the spacing between the footholds, and the strength of walker-foothold binding are carefully optimized to ensure that the walkers remain continuously bound to at least one foothold while transitioning to the next foothold within a relatively short time, typically on the order of seconds ^22^. These stepping times lower those of previous DNA walker systems by at least an order of magnitude ^23^. However, they are found to be capped at $\sim 1$s per transition, underscoring a bottleneck that limits the potential of DNA nanomachines to approach the efficiency of natural motor proteins ^16^.

In this work, we address this limitation by modifying the DNA-based track to reduce the phase space the walker explores before transitioning from one foothold to another. By introducing a double-stranded (ds) DNA ‘tail’ to the footholds, steric interactions facilitate in-plane rotation of the walkers, suggesting a reduced phase space which achieves a fourfold reduction in stepping time. This enhancement sets a new benchmark for the speed and efficiency of DNA nanotechnology, showcasing how more precise control of local DNA mechanics may unlock faster, more reliable molecular systems and expand the functional capabilities of dynamic DNA-based devices.

## Results

### Tethered motion as a diffusion process

To characterize the stepping time, binding will be modeled as a chemical reaction between the center of the last base pair of the ds region of the walker-foothold complex (what we will refer to as the ‘free end’ of the complex) and a fixed target on the substrate (the base of the next foothold). A schematic of this process is provided in Fig. 1. In this framework, the short ss region that makes initial contact with the next foothold (referred to as ‘toehold’), is accounted for within the definition of a binding distance $\epsilon$. This coarse-grained treatment has been shown to effectively capture key kinetic characteristics of cartwheeling DNA walkers while enabling an analytical characterization of the stepping time ^22^.

**Fig. 2:**
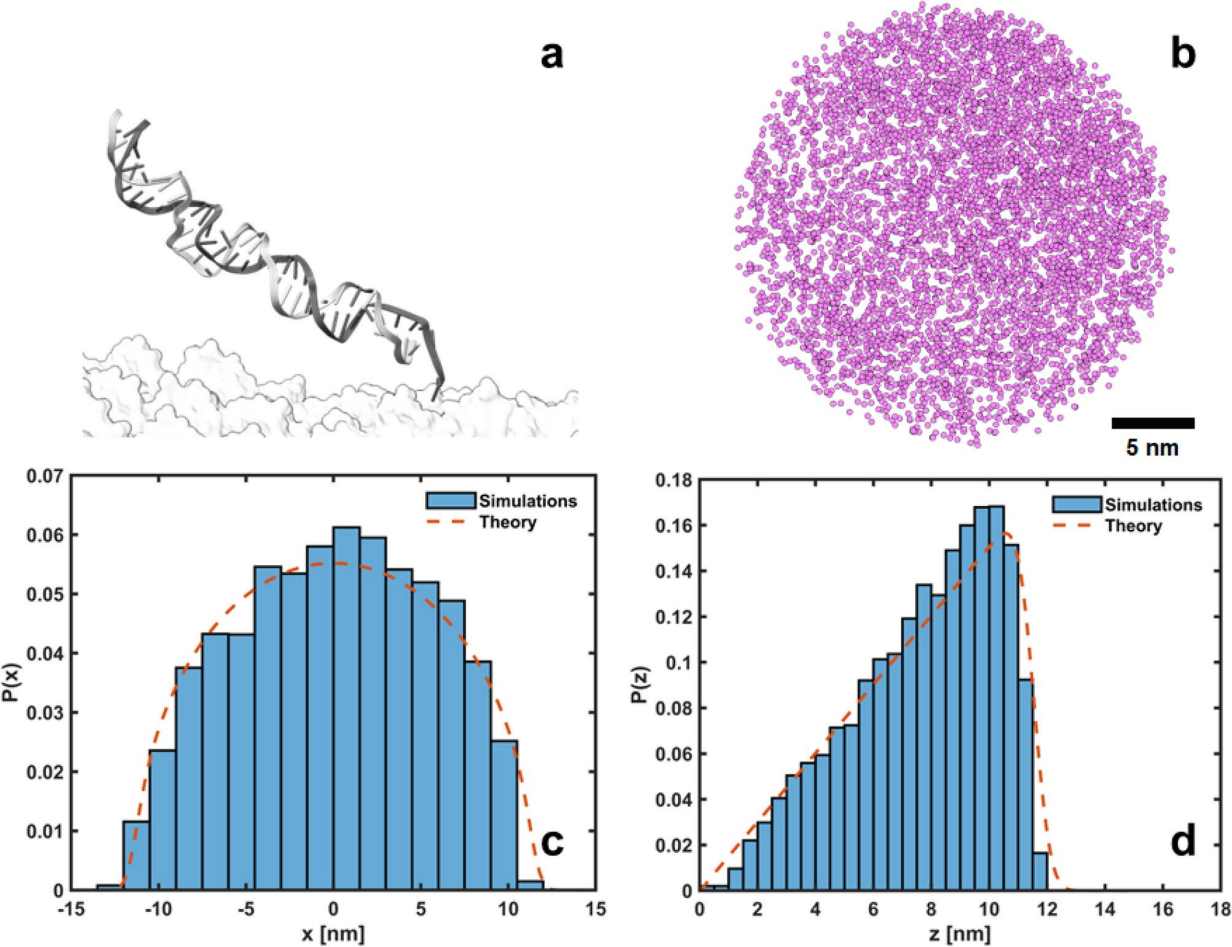
(**a**) Detail of the simulated walker-foothold complex (36 bps long). (**b**) In-plane (x-y) projection of simulated positions of the free end of the complex. (**c**) Simulated *x* positions of the free end of the complex averaged over *y* and *z* (histogram). Average probability density *P*(*x*) marginalized over *y* and *z* (dashed line). (**d**) Idem to (**c**) along the *z* coordinate (out-of-plane). $L=11.5$ nm, $\beta k=5$ $nm^{-2}$. See Methods for oxDNA simulation protocol.

Due to the short length of the DNA walkers (smaller than the persistence length of double-stranded DNA ^24^), the walker-foothold complex (Fig. 2a) behaves as a structurally constrained system and its free end samples conformations along a hemispherical shell^25^ (see Fig. 2b–d), in contrast to the behavior of longer molecules, which resemble grafted Gaussian chains ^22,26,27^. Taking as the center of our system of coordinates the tethering point of the foothold (the nucleotide closest to the platform), the probability of finding the free end of the complex at a location $\vec {r}=(x,y,z)$ can be written as

1 $$\begin{aligned} P(\vec {r})=ze^{-\frac{\beta k}{2}(\Vert \vec {r}\Vert -L)^{2}}, \end{aligned}$$

where $\Vert \vec {r}\Vert$ is the norm of $\vec {r}$, $\beta =1/k_{B}T$ is the reciprocal of the thermal energy, *L* is the mean length of the complex (close to its contour length - 0.31 nm per base-pair ^28^) and *k* is a universal stiffness parameter (see Fig. S1). Equation (1) was selected empirically to provide a functional form that approximates the simulated data. However, its foundation lies in polymer dynamics and statistical mechanics: the Boltzmann-weighted harmonic potential characterizes confined diffusion where a particle is biased to remain near the surface ^29^ $\Vert \vec {r}\Vert =L$, and the inclusion of *z* as a prefactor (inspired by the Rayleigh distribution ^30^) accounts for the bias introduced by tethering the foothold to a surface. Upon normalization on $z>0$, the distribution introduced in Eq. (1) accurately captures the constrained dynamics of the free end of the complex, concentrating the probability mass near the spherical shell and aligning well with simulated results (see Fig. 2b–d).

Since by definition binding can only take place when the target and the free end of the complex are at a distance of $\epsilon$, it will be convenient to construct a mean-field radial probability distribution for the distance $\rho$ between the target and this free end to reduce the dimensionality of our system^27,31^. Switching to spherical polar coordinates $(\rho ,\theta ,\phi )$ centered on the target, which we may considered to be placed at (*a*, 0, 0) without loss of generality, as $x=a+\rho \sin \theta \cos \phi$, $y=\rho \sin \theta \sin \phi$, $z=\rho \cos \theta$, the propagator for the position of the free end can be written as

2 $$\begin{aligned} P(\rho ,\theta ,\phi )=\rho \cos \theta e^{-\frac{\beta k}{2}(\sqrt{a^{2}+2a\rho \sin \theta \cos \phi +\rho ^{2}}-L)^{2}}. \end{aligned}$$

Integrating this expression over the two angles $\theta \in [0,\pi /2]$, $\phi \in [0,\pi ]$, a radial probability density about the target receptor $P_{eq}(\rho )$ can be derived, which may be associated to an effective radial potential ^27^ $U(\rho )=-\beta ^{-1}\ln {[\rho ^{2}P_{eq}(\rho )]}$. The dynamics of the free end can then be modeled as those of a one-dimensional particle (whose position is tracked by $\rho$) diffusing in a landscape $U(\rho )$, which may react with the target when $\rho =\epsilon$ with a probability proportional to the sequence-dependent reactivity $\kappa$ ^22,32^. In this scenario, the mean stepping time (i.e., the time it takes for this particle to react with the target) can be calculated using Szabo’s relation^33^

3 $$\begin{aligned} \tau =\frac{1}{D}\int _{\epsilon }^{+\infty } e^{\beta U(\rho )}\Big [\int _{\rho }^{+\infty }d\rho 'e^{-\beta U(\rho ')}\Big ]^{2}d\rho +\frac{ e^{\beta U(\epsilon )}}{\kappa }, \end{aligned}$$

where *D* is the diffusion coefficient of the free end of the complex, and the initial positions of the free ends are assumed to follow a Boltzmann distribution defined by $U(\rho )$. The first term of this equation represents the time it takes for the first encounter to take place, while the second term accounts for the time spent in subsequent rebounds until a successful binding event occurs ^34,35^.

Due to the complexity of DNA hybridization and toehold-mediated strand displacement, highly imperfect binding (small $\kappa$) is the norm in bimolecular DNA strand reactions on a substrate ^22,35^. In particular, as $\tau$ is dominated by any regions where $U(\rho )\rightarrow \infty$, expanding Eq. (3) for small $\rho$ suggests that the ratio of the reaction-limited (rightmost) to the diffusion-limited (leftmost) contributions to $\tau$ is of the order of^22,27^ $D\sqrt{\beta k}/\kappa$. For the DNA walkers introduced by Li et al. ^20^ ($D\sim 50$ $\mu \hbox {m}^{2}$/s, $\kappa \sim 10$ nm/s according to prior simulations ^22^), this term is $\sim$ $10^{6}$. As a result, the stepping time is predominantly determined by $\kappa$ (assumed constant if the sequences of the footholds and walkers remain unchanged) and the energy landscape $U(\rho )$. This article aims to explore potential modifications to the nucleic acid ‘track’ along which the walker moves, with the goal of reducing the stepping time by reshaping $U(\rho )$. For simplicity, we will neglect the effects of volume exclusion from neighboring footholds, expecting this factor to have minimal impact on relative comparisons.

### Introducing bias via steric interactions

#### Pseudo-rotational dynamics

**Fig. 3:**
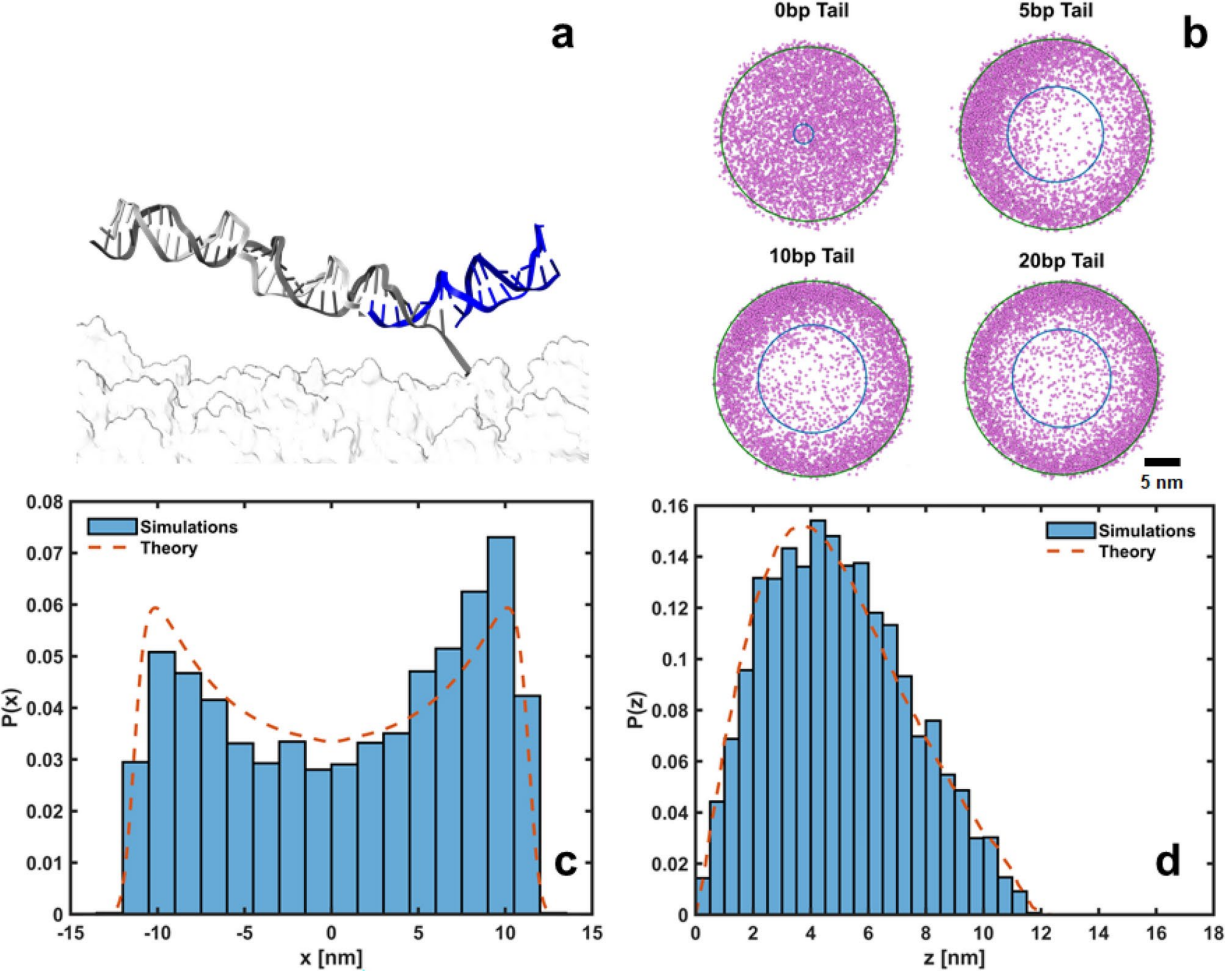
(**a**) Detail of the simulated tailed (12 bps long) walker-foothold complex (36 bps long). Note that a region of the foothold (besides its tail) must be made ds to allow for the tail to stay hybridized to the foothold. (**b**) In-plane (x–y) projection of simulated positions of the free end of the complex (36 bps long) for different tail lengths. The lines represent the best-fit rings, defined by an outer radius ($R_{max}$) and an inner radius ($R_{min}$), optimized to minimize the enclosed area while containing 90% of the data. (**c**) Simulated *x* positions of the free end of the tailed complex averaged over *y* and *z* (histogram). Average probability density $P_{tail}(x)$ marginalized over *y* and *z* (dashed line). (**d**) Idem to (**c**) along the *z* coordinate (out-of-plane). $L=11.5$ nm, $\beta k=5$ nm$^{-2}$, $\beta k'=0.75$ nm$^{-2}$, $z_{max}=12.5$ nm.

Inspired by previously developed DNA origami nanorotors where in-plane rotation of DNA bundles is driven by steric interactions ^36,37^, we extend the footholds of our track by the inclusion of a ds DNA ‘tail’ (see Fig. 3a and Fig. S2a) to introduce steric hindrance at smaller lengths. Different tail lengths were studied, suggesting that a minimal length of $\sim$ 6 base-pairs (bps) is needed to reduce out-of-plane motion consistently (see Fig. 3b and Fig. S2d), in agreement with mechanical cantilever models^38^ (see Supplementary Materials S2).

To estimate the stepping time in this scenario, we first need to derive an expression for the energy landscape $U(\rho )$ in the presence of a tail. This landscape governs the dynamics of the free end by accounting for the steric and energetic constraints introduced by the tail, which modify the accessible configurations and proximity to the next foothold. Based on the expressions derived for a Gaussian chain within a slit by means of the method of images ^27,39^, we will model this confined motion by introducing a probability distribution that accounts for both the natural (harmonic) motion of the free end of the complex, and the steric confinement imposed by the substrate and the tail,

4 $$\begin{aligned} P_{tail}(\vec {r}) = e^{-\frac{\beta k}{2}(\Vert \vec {r}\Vert - L)^2} \sum _{n=1}^{+\infty } \sin \Big (\frac{n\pi z}{z_{\text {max}}}\Big ) e^{-\frac{\beta k}{2} \Big (\frac{n\pi }{[\beta k']z_{\text {max}}}\Big )^2}, ~~0\le z\le z_{max} \end{aligned}$$

The Fourier-inspired summation introduces the effects of steric confinement along the *z*-axis, where the sine terms ensure that the probability vanishes at the boundaries $z = 0$ and $z = z_{max}$, mimicking a slit-like geometry ^27^. The exponential damping factor within the series reflects the increasing energetic cost of higher-order oscillatory modes, ensuring that the distribution is dominated by smooth, low-frequency motions. The fitting parameter $k'$ reflects the effective stiffness of the tail, modulating the walker’s ability to sample higher-frequency oscillatory modes. A higher $k'$ reduces the energetic cost of these modes, enabling the walker to explore configurations closer to the boundaries of the confined space. Conversely, a lower $k'$ strongly suppresses higher-order modes, restricting the walker’s motion to smoother, lower-energy configurations near the center of the ‘slit’. For sufficiently long tails, $k'$ becomes independent of the tail’s length. However, both $z_{max}$ and $k'$ are influenced by the length of the walker-foothold complex (see Fig. 3 and Fig. S3), as longer complexes possess greater entropy and exhibit increased deflection, enabling the walker to sample larger heights (see Supplementary Materials S2).

Figure 3c shows the average probability density $P_{tail}(x)$ marginalized over *y* and *z* for a tail length of 12 bps. Due to the axisymmetry of the system, identical results are observed for the average probability density $P_{tail}(y)$ marginalized over *x* and *z*. Figure 3d shows the average probability density $P_{tail}(z)$ marginalized over *x* and *y*, suggesting that the tail effectively prevents the walker from adopting a vertical orientation. The vertical orientation is the most common without a tail (see Fig. 2c, d), as the flexibility of the tethering point allows the walker to sample all orientations uniformly, favoring configurations that maximize the distance between its free end and the substrate^25^. Simulations confirm that Eq. (4) effectively captures the dynamics of the free end in the presence of a tail, supporting the idea of a relatively stiff tail. The implications of this confinement on the stepping time will be further explored in “Derivation of mean stepping times”.

#### Pseudo-curvilinear dynamics

**Fig. 4:**
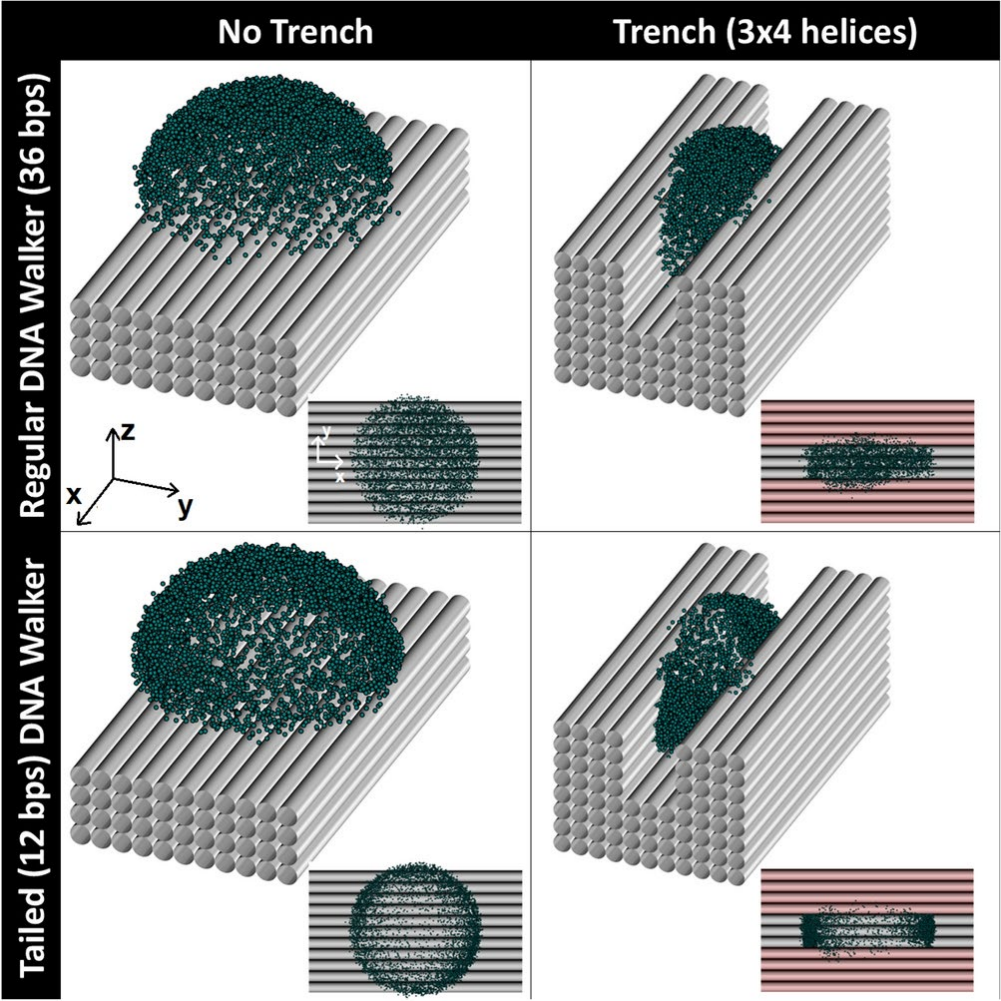
Schematic of the systems studied in this work. Symbols represent simulated positions of the free end of the walker-foothold complex. When a trench is included, this trench was built 4 helices tall ($\sim 8$ nm^40^) by 3 helices wide ($\sim 6$ nm), narrow enough to confine the lateral motion of molecules larger than $\sim 20$ bps. To study the confinement resulting from the trench, a 36-bps-long walker-foothold complexes is the focus of this study. Results for a smaller molecule (21 bps) with and without a tail are reported in Supplementary Materials.

Confinement reduces the sampling of undesired conformations, potentially decreasing the stepping time (see “Derivation of mean stepping times”). Building on this concept, we investigated the effects of further restricting the walker’s motion by introducing a trench-like geometry parallel to the *x* axis of our system of coordinates (see Fig. 4 and Fig. S4). This design confines one of the walker’s in-plane degrees of freedom, guiding its motion predominantly along a one-dimensional arc. The trench limits lateral movement while allowing forward progression, narrowing the range of accessible configurations and focusing the walker’s dynamics along a defined track. To this end, we modified the previously used DNA origami substrate and added two additional sets of parallel DNA bundles, creating a trench of 3 helices wide and 4 helices high.

**Fig. 5:**
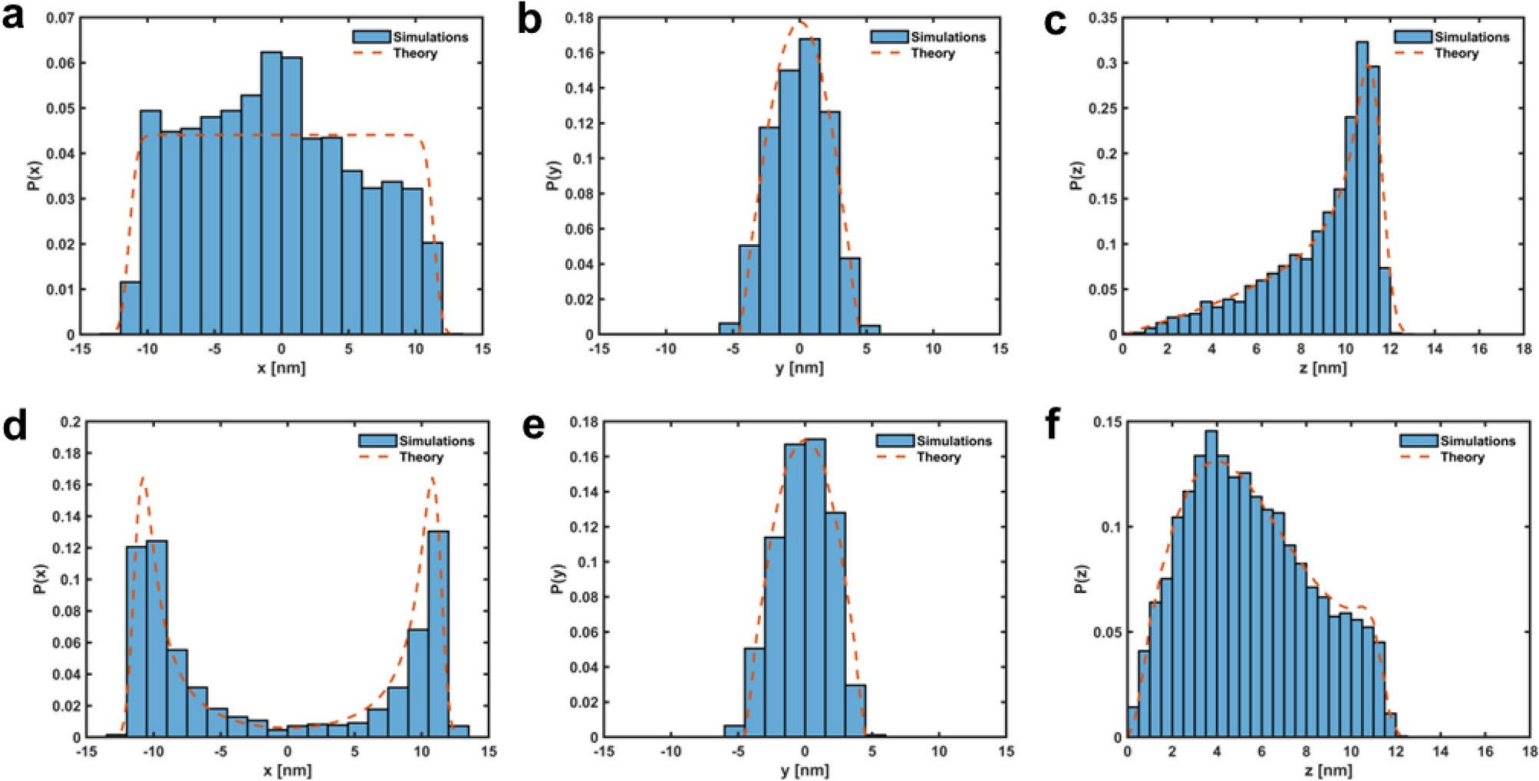
(Top) Simulated *x* (**a**), *y* (**b**), and *z* (**c**) positions of the free end of the walker-foothold complex (36 bps) averaged over the remaining degrees of freedom in presence of a trench (histogram). Average probability densities $P_{trench}$ marginalized over the remaining degrees of freedom (dashed lines). (Bottom) Simulated *x* (**d**), *y* (**e**), and *z* (**f**) positions of the free end of the tailed (12 bps) complex (36 bps) averaged over the remaining degrees of freedom in presence of a trench (histogram). Average probability densities $P_{combined}$ marginalized over the remaining degrees of freedom (dashed lines). $\beta k=5$ nm$^{-2}$, $\beta k'=0.75$ nm$^{-2}$, $L=11.5$ nm, $z_{max}=12.5$ nm, $h_{max}=9$ nm. Note that a shoulder is observed in (**f**), reflecting the bistable nature of our combined tail-trench system. This shoulder represents the configurations sampled during transitions among states.

To model the motion of the free end of the complex in the presence of a trench, we adapt the framework previously established for the tailed footholds. Specifically, the trench imposes additional constraints along the *y*-axis, which can be described by a Fourier-inspired probability distribution

5 $$\begin{aligned} P_{trench}(\vec {r}) = ze^{-\frac{\beta k}{2}(\Vert \vec {r}\Vert - L)^2} \sum _{m=1}^{+\infty } \cos \Big (\frac{m\pi y}{h_{\text {max}}}\Big ) e^{-\frac{\beta k}{2} \Big (\frac{m\pi }{[\beta k'']h_{\text {max}}}\Big )^2}, ~~z\ge 0,~~ -\frac{h_{max}}{2}\le y \le \frac{h_{max}}{2}, \end{aligned}$$

where $h_{max}$ is the width of the trench and $k''$ is a stiffness parameter similar to that introduced in the steric confinement model. Simulations suggest that the dynamics are better captured by a simplified version of this distribution (see Fig. 5a–c),

6 $$\begin{aligned} P_{trench}(\vec {r}) = ze^{-\frac{\beta k}{2}(\Vert \vec {r}\Vert - L)^2} \cos \Big (\frac{\pi y}{h_{\text {max}}}\Big ), ~~z\ge 0,~~ -\frac{h_{max}}{2}\le y \le \frac{h_{max}}{2}, \end{aligned}$$

where the cosine term represents the confinement introduced by the trench walls, and the lack of higher-order terms reflects an effective reduction in accessible modes along *y*.

Figures 5a–c illustrate the effects of trench-induced confinement on the probability density of the free end of our walker-foothold complex. In comparison to the tailed foothold scenario, the trench significantly sharpens the distribution along the *y*-axis, as seen in Fig. 5b, indicating reduced lateral mobility. Figure 5c reveals an intriguing phenomenon: as the walker experiences steric repulsion from the substrate, it preferentially adopts configurations where its free end is positioned at higher values of *z*. This shift in sampling dynamics can be attributed to the interplay between steric forces and the harmonic tethering potential, which naturally seeks to confine the walker near its equilibrium contour length *L*. However, the trench breaks the axisymmetry of the system, favoring configurations where the walker extends upward to avoid interactions with the substrate. This upward bias diminishes the advantages of confinement, as it reduces the walker’s effective proximity to the next foothold, partially counteracting the intended reduction in stepping time by increasing the average distance the walker must traverse to bind. The implications of this confinement on the stepping time will be further explored in “Derivation of mean stepping times”.

#### Bi-stable dynamics

Building on the previous observations, a natural next step is to explore what happens when both confinement strategies, footholds with ds tails and trench-like geometries, are combined (see Fig. 4). Placing ds-tailed footholds within the trench restricts the walker’s motion both vertically and laterally (see Fig. 5e, f). Combined with the inherent stiffness of the walker, this dual-confinement significantly limits the walker’s accessible configurations, introducing bi-stable dynamics where the walker alternates between two constrained conformations within the trench (see Fig. 5d). This bi-stability could be anticipated from our mathematical models of the walker’s probability distributions under tail and trench constraints. Combining the probabilities from Eqs. (4) and (6),

7 $$\begin{aligned} P_{combined}(\vec {r})= & e^{-\frac{\beta k}{2}(\Vert \vec {r}\Vert - L)^2}\cos \Big (\frac{\pi y}{h_{\text {max}}}\Big ) \sum _{n=1}^{+\infty } \sin \Big (\frac{n\pi z}{z_{\text {max}}}\Big ) e^{-\frac{\beta k}{2} \Big (\frac{n\pi }{[\beta k']z_{\text {max}}}\Big )^2}, ~~0\le z\le z_{max},~~\nonumber \\ & \quad -\frac{h_{max}}{2}\le y \le \frac{h_{max}}{2}, \end{aligned}$$

is found to be in great agreement with simulations (see Fig. 5d–f), reinforcing the robustness of our empirical framework.

### Derivation of mean stepping times

**Fig. 6:**
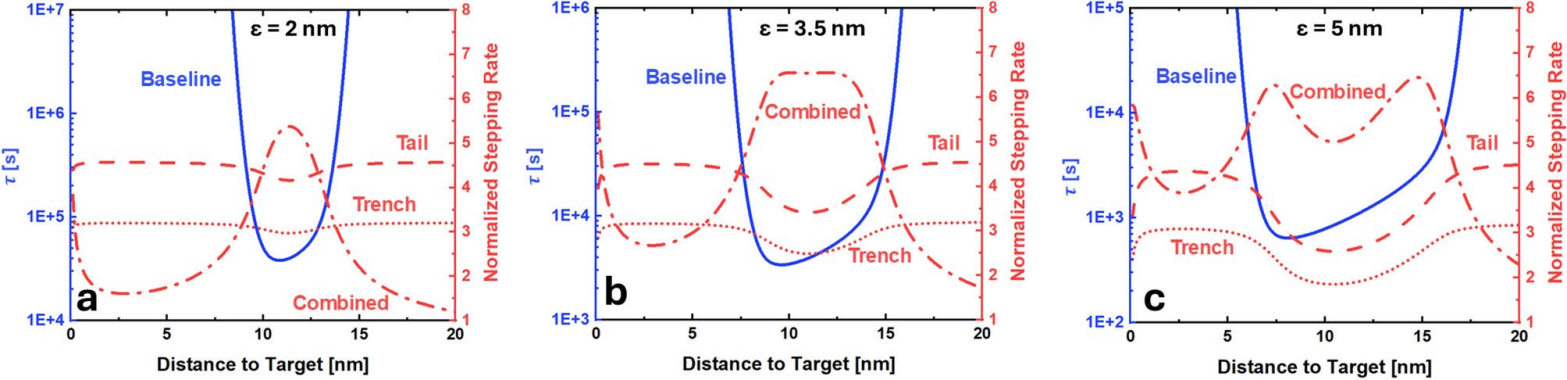
Mean stepping time (‘baseline’, $\kappa =1$ nm/s) of a walker-foothold complex (36 bps) in absence of tail and trench (solid, blue) estimated from our fitted distributions using Eq. (3) in the reaction-limited regime as a function of the distance between the tethering point and the target, *a*. Normalized stepping rates (with respect to the baseline) estimated from our fitted distributions using Szabo’s equation for a tailed foothold (12 bps tail, 36 bps complex, dash, red), a trench (36 bps complex, dots, red), and a combined tail-trench system (12 bps tail, 36 bps complex, dash-dot, red). The ssDNA toeholds employed by Li et al. ^20^ that allow the walker to bind to the next foothold consist of 8 nts. These strands exhibit a contour length of $\sim 5$ nm ^43^, a Kuhn length of 1.6 nm ^44^ and (by means of worm-like chain estimates ^45^) an end-to-end distance of 3.4 nm. We then consider $\epsilon =2$ (**a**), 3.5 (**b**), and 5 (**c**) nm for our analysis. In all calculations, $D=50$ $\upmu \hbox {m}^{2}$/s was taken, leading to negligible contributions of the diffusion-limited term to $\tau$.

In the previous sections, we derived the probabilities of finding the free end of the walker-foothold complex in different scenarios: the baseline case described by Li et al. (*P*), the addition of a ds tail to the foothold ($P_{tail}$), the placement of the DNA track within a trench-like DNA origami platform ($P_{trench}$), and the combination of tailed footholds within the trench ($P_{combined}$). By converting these probabilities to spherical polar coordinates centered on the target and integrating over the two angles, we obtain radial probability densities about the target. These densities enable us to estimate the stepping time as a function of the target position *a* using Szabo’s relation, as described in “Tethered motion as a diffusion process”. Figure 6 presents a comparison of numerically evaluated stepping rates (i.e., inverse of the stepping times) normalized against the baseline scenario (without a trench or tail) as a function of *a* for a walker-foothold complex of length 36 bps (see Fig. S4 for similar results on a 21 bps complex).

The observed results align well with our expectations. Introducing a tail improves binding by approximately a factor of 4, demonstrating that confining the vertical motion of the foothold and converting diffusive walkers into rotors is an effective strategy to achieve faster communication. This effect becomes less pronounced as we increase the length of the toehold (increase $\epsilon$), reducing the effect of the energetic landscape by allowing binding to take place at a wider phase space. Trenches are less effective than the tails because they force the walker to sample vertical positions more frequently. While they are better than no confinement at all (as they reduce the overall entropy of the system), the added cost of using more DNA strands to construct the trenches outweighs their benefits. Additionally, incorporating a trench will not be advantageous for small molecules ($<20$ bps), where interactions with the walls will be less prominent.

Integrating both strategies (tail and trench) significantly reduces the system’s entropy, effectively transforming the walker-foothold system into a bistable one characterized by two distinct stable states (local energy minima) separated by an energy barrier ^41^ (see Fig. 5d). To the best of our knowledge, this is the smallest synthetic DNA nanostructure which exhibits such bistable mechanism. Due to the intrinsic flexibility of the molecule ^42^, the walker can still transition between the two states (see Fig. S5 and Animation S1). However, our estimates assume that the walker’s initial position follows a Boltzmann distribution. In practice, the walker is more likely to start on the side opposite to the foothold, what would significantly increase the actual stepping time compared to the theoretical prediction we derived based on Szabo’s equation (which averages over all possible initial positions).

## Discussion

In this work, we conducted a theoretical and computational study of molecular communication in nanoscale systems ^46^, focusing on DNA walkers as model components. By introducing and analyzing specific strategies relying on steric interactions ^47^, such as the addition of tails to footholds and the use of trench-like geometries, we demonstrated how these mechanisms alter the energy landscape of DNA walkers, fine-tuning their motion and improving communication capabilities. This approach relies on steric interactions and is thus sequence independent; therefore, our strategies do not interfere with the available walkers’ (sequence)design-space, avoiding potential cross-talk with the DNA track or other DNA components present. Our results underscore the potential of structural motifs to improve molecular transport and control in nanoscale devices, contributing to the ongoing development of DNA-based nanomachines ^48,49^.

While our primary focus was on enhancing the communication speed in DNA walker systems, an interesting contribution of this study is the development of a combined tail-and-trench mechanism which significantly reduces the entropy of the system by confining the walker’s accessible configurations. This dual strategy transforms the walker-foothold system into a bistable one characterized by two distinct stable states separated by an energy barrier, offering opportunities for selective pathway control and motion modulation. Such designs could enable programmable nanoscale logic gates, where the walker’s motion depends on steric constraints, or serve as biosensing platforms. For instance, the walker could be directed along distinct paths in response to the presence or absence of analytes, opening avenues for targeted molecular diagnostics and smart therapeutic delivery systems.

Potential improvements to our model include refining the representation of DNA toeholds, which are currently simplified into a binding distance parameter $\epsilon$, and incorporating volume exclusion effects between adjacent footholds for a more accurate depiction of steric interactions. Our simulations, grounded in the well-validated oxDNA model, provide a robust theoretical framework that offers predictive insights for future experimental validation. These findings highlight the potential of structural confinement strategies to enhance DNA walker efficiency, paving the way for more advanced molecular transport systems in DNA nanotechnology.

## Methods

### Simulations

DNA origami devices were designed on a square lattice using caDNAno(2.4) ^50^. The scaffold strand length for all versions was chosen to be similar to commonly used lengths ^51,52^. Single-stranded poly-T extensions were not included ^53,54^. The design files are available at nanobase.org ^55^. CaDNAno designs were converted into oxDNA-compatible formats using the tacoxDNA Python package ^56^. To ensure proper base-pairing and minimize steric clashes, the structures underwent minimization on the oxDNA.org server ^57^. Following minimization, the structures were equilibrated using Monte Carlo (MC) simulations in oxDNA ^58^. This stage employed a CPU backend in double precision, with maximum backbone forces set to 5 (near) and 10 (far). The DNA2 ^59^ interaction model was applied at a 1 M salt concentration, and simulations were run in the NVT ensemble with translation and rotation delta parameters set to 0.22. A total of 10,000 steps were simulated at 283 K ($10^\circ \hbox {C}$) to ensure initial relaxation. After equilibration, molecular dynamics (MD) simulations were conducted to further refine the configurations. These simulations used the CPU backend with a Langevin thermostat, maintaining a temperature of 283 K. A timestep of 0.002 oxDNA time units ($\sim$6.06 fs) was employed for a total of 20 million steps. For the production phase, MD simulations were implemented using the CUDA backend ^60^, with mixed precision and adaptive cell optimization at a salt concentration of 0.5 M consistent with experiments ^61^. This high ionic strength (associated to a Debye screening length of $\sim$ 0.4 nm) renders electrostatic interactions negligible at longer ranges, a standard assumption in DNA nanotechnology, as high salt contents are essential for the stability of DNA origami ^43,58,61^. A timestep of 0.001 oxDNA time units ($\sim$3.03 fs) was chosen for higher temporal resolution, with the thermostat switched to the John algorithm to emulate Brownian motion. Key parameters included 103 Newtonian steps and a diffusion coefficient of 2.5. Periodic outputs of configurations and energies were generated every $10^{5}$ steps. Simulations were run for approximately 2 $\mu$s of simulated dynamics ($\sim 6\times 10^{8}$ steps). The resulting trajectories and configurations were analyzed using custom-made Python scripts.

### Post-processing and data characterization

All post-processing and data characterization was performed by means of custom-made codes in Python developed in our lab. Fitting of data to rings (Fig. 3b, Fig. S2) was done by implementing an optimization process to determine the smallest annulus enclosing $90\%$ of the data using the brute force optimization method with multiprocessing. Numerical integration of the probabilities *P*, $P_{tail}$, $P_{trench}$ and $P_{combined}$ was done using the nquad function for numerical stability. The constraints resulting from introduction of the walls ($-h_{max}/2\le y \le h_{max}/2$) and the tail ($0\le z\le z_{max}$) are imposed in the integration domain in angular space by means of Heaviside functions ^31,35^, leading to the effective radial potentials $U(\rho )$ used in Eq. (3). Infinite sums were approximated using a finite truncation parameter $N=20$, ensuring a balance between computational efficiency and accuracy.

## Supplementary Information

Supplementary Information.

## Data Availability

Design files are available at nanobase.org.
