# Supplementary material for: Enhancing the speed of DNA walkers through soft confinement
Source: Sci Rep. 2025 Mar 19;15:9450. doi: 10.1038/s41598-025-93269-x (PMC11923261; doi:10.1038/s41598-025-93269-x)
Supplement: Supplementary file 1 — Supplementary Information. [file 41598_2025_93269_MOESM1_ESM.pdf]

Enhancing The Speed of DNA Walkers Through Soft Confinement

Mathew Osaretin Ogieva, Wolfgang Georg Pfeifer, Sebastian Sensale

Supplementary Materials

S1. Statistics of Walker-Foothold Complexes

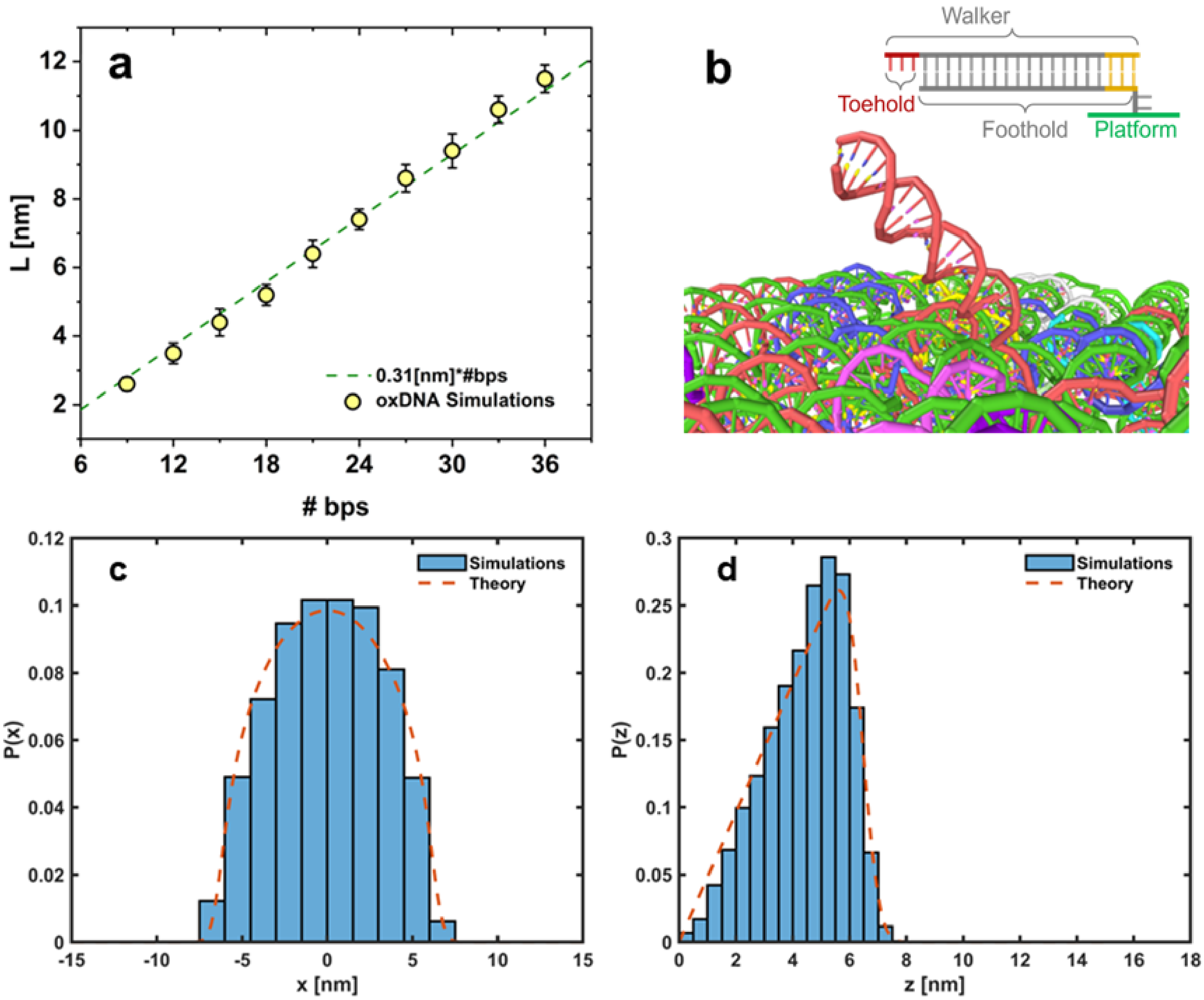

Figure S1. (a) Fitting of simulated free end dynamics of walker-foothold complexes with different numbers of bps (from 9 to 36 every 3) to **Eq. 1** provides values of L compatible with 0.31 nm per bp. For all fittings, $\beta k = 5\ nm^{-2}$ was found to be in great agreement with simulated data. (b) Detail of a simulated walker-foothold complex (21 bps long). (INSET) Schematic of the walker-foothold junction. (c) Simulated x positions of the free end of the (21 bps long) complex averaged over y and z (histogram). Average probability density $P(x)$ from Eq. (1) marginalized over y and z (dashed line). (d) Idem to (c) along the z coordinate (out-of-plane).

For completeness, we mention that the average probability density $P(x)$ marginalized over y and z is given by

$$P(x) = \frac{\int_0^{+\infty}\int_{-\infty}^{+\infty} P(x,y,z)dydz}{\int_{-\infty}^{+\infty}\int_0^{+\infty}\int_{-\infty}^{+\infty} P(x,y,z)dydzdx}, \tag{S1}$$

and idem for the other averaged densities.

S2. Statistics of Tailed Walkers

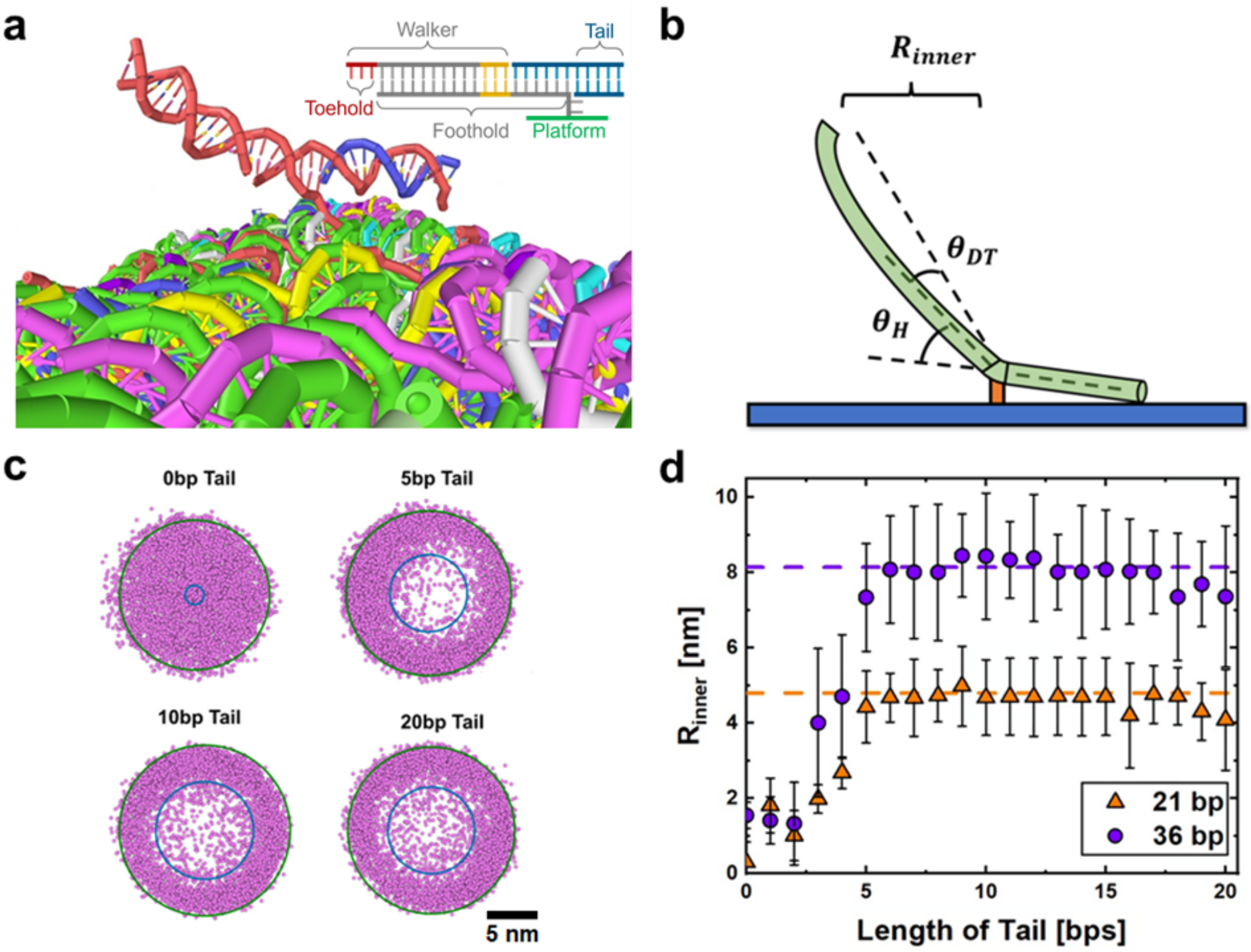

Figure S2. (a) Detail of a simulated walker-foothold complex (21 bps long) with a tail of length 12 bps. (INSET) Schematic of the walker-foothold-tail junction. (b) Diagram of angles and measurements involved in our mechanical cantilever model. (c) The lines represent the best-fit rings, defined by an outer radius (R_{max}) and an inner radius (R_{min}), optimized to minimize the enclosed area while containing 90% of the data. (d) Comparison between simulated (symbols) and theoretically estimated (Eq. S2, dashed lines) inner radii for two different walker-foothold complexes and multiple ds tail lengths. Error bars were calculated from the deviation of fitting to rings which include 90%, 92.5%, and 95% of the data.

As the tail cannot penetrate the surface, its introduction (**Figure S2a**) restricts the angles the walker-foothold complex can sample with respect to the plane of the platform. To prove this, we will consider an effective maximum angle θ_H between the tail and the axis of the walker-foothold complex independent of molecule (and tail) length resulting from the mechanical properties of their junction (see **Figure S2b**). This angle will be taken as a fitting constant. The walker-foothold complex is short (and therefore quite rigid); however, it still has a finite persistence length L_p. Considering its pivot point to be fixed and modeling the rest of the molecule (walker+foothold) as a cantilever of length L (not including the tail), the free end of the complex will exhibit a deflection of approximately[1] $\Delta z \sim L^2/2L_p$, equivalent to an angle $\theta_{DT} \sim L/2L_p$. Currently, there are two nucleotides (nts) between the foothold and the DNA origami platform, with a length of approximately 0.64 nm per nucleotide[2]. For dsDNA, where the length per base pair is 0.31 nm, it takes ~ 5 bps to reach this height. Thus, a tail length of 6 bps or more is required to consistently influence the dynamics in this scenario (see **Figure S2d**).

For a large enough tail, as the tail cannot penetrate the surface, the angle between the platform and the tail (clockwise in **Figure S2b**) will be small. Thus, it will be rare for the free end of the complex to sample angles larger than $\theta_H + \theta_{DT}$ (where

the tail is horizontal). This maximum angle implies a minimum radius (**Figures S2c-d**)

$$R_{\text{inner}} = L\cos(\theta_H + \theta_{DT}) = L\cos(\theta_H + \frac{L}{2L_p}). \tag{S2}$$

For small molecules (units to tens of bps), the persistence length of dsDNA is length dependent[3]; in particular,

$$L_p = 1.14 \cdot \#\text{bps} + 30.30[nm] \tag{S3}$$

for the oxDNA force field[4]. Fitting R_{inner} to simulations of walker-foothold complexes with 21 and 36 bps for tails of lengths 7 to 20 bps, an effective angle $\theta_H \sim 39°$ was found (**Figure S2d**).

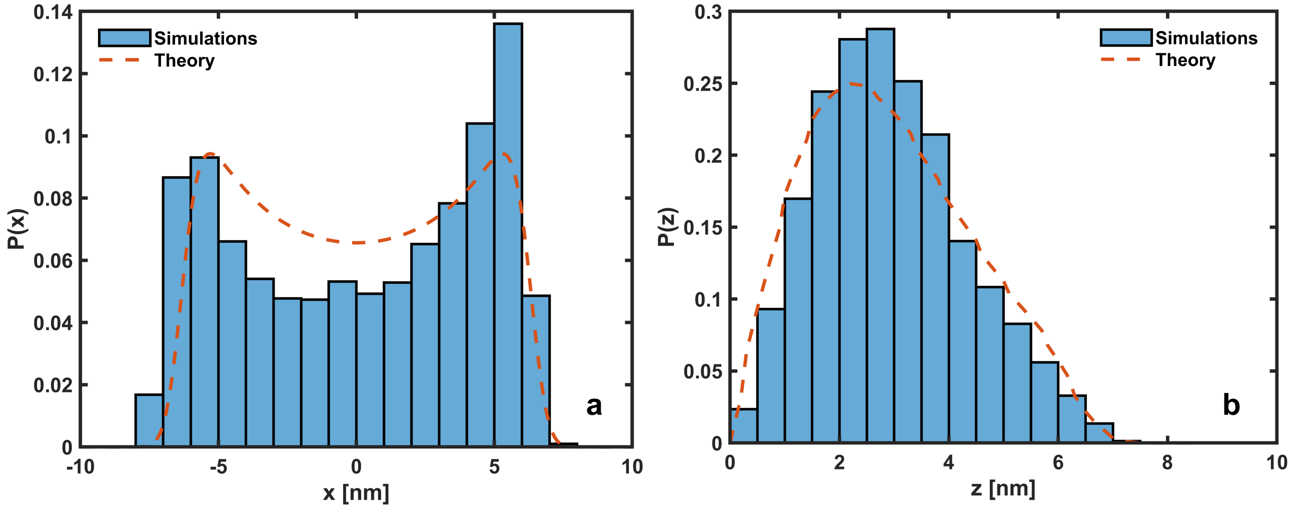

Figure S3. (a) Simulated x positions of the free end of the tailed (12 bp tail) walker-foothold complex (21 bps long) averaged over y and z (histogram). Average probability density $P_{tail}(x)$ marginalized over y and z (dashed line). (b) Idem to (b) along the z coordinate (out-of-plane). $L = 6.4$ nm, $z_{max} = 8.8$ nm, $\beta k = 5\ nm^{-2}$, $\beta k' = 1.25\ nm^{-2}$.

S3. Mean Stepping Times for 21-bps-long Walker-Foothold Complexes

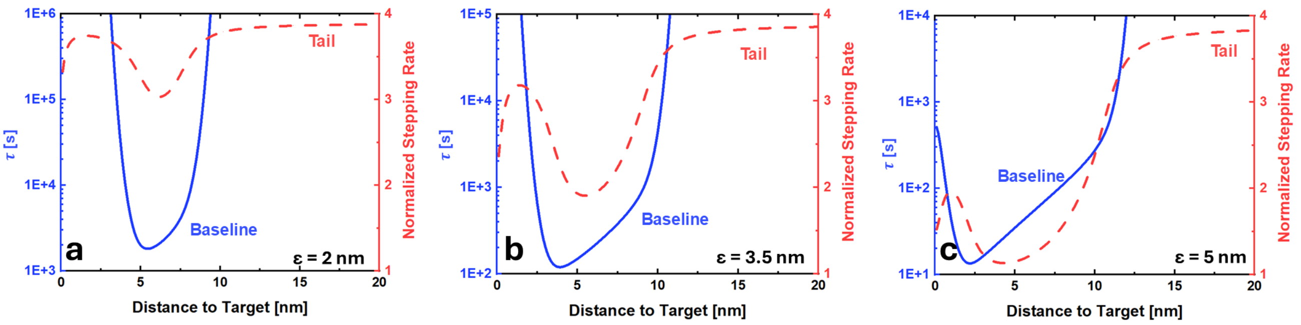

Figure S4. Mean stepping time ('baseline', $\kappa = 1$ nm/s) of a walker-foothold complex (21 bps) in absence of tail and trench (blue), estimated from our fitted distributions using Szabo's equation, as a function of the distance between the tethering point and the target, a. Normalized stepping rates (with respect to the baseline) estimated from our fitted distributions using Szabo's equation for a tailed system (12 bps tail, 21 bps complex, dash, red). $\varepsilon = 2$ nm (a), $\varepsilon = 3.5$ nm (b), $\varepsilon = 5$ nm (c). In all calculations, $D = 50\ \mu\text{m}^2$/s was taken, leading to negligible contributions of the diffusion-limited term to τ.

S4. Transforming DNA Walkers into Bistable Nanostructure

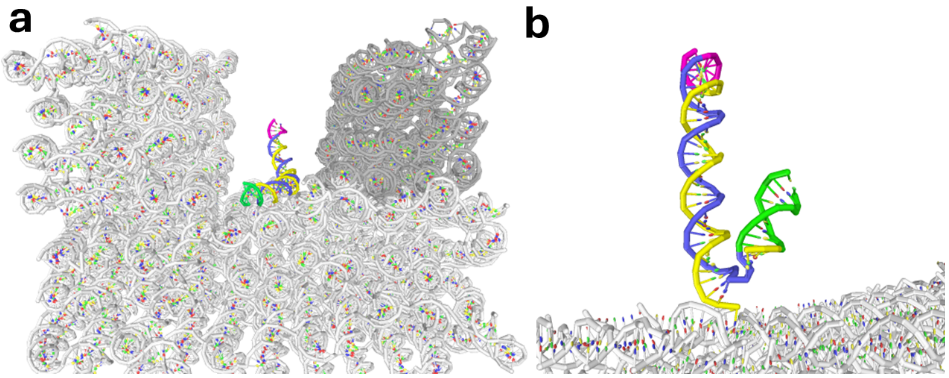

Figure S5. (a)A tailed (12 bps tail) DNA walker (36 bps) inside our trench. (b) Snapshot of the configuration of the walker as it moves from one side to the other within this system. See **Animation S1** for an example of this transition. Magenta and green colors were added to ease the tracking of the ends of the walker-foothold (magenta) and tail (green) components.

Animation S1

Example of a simulated transition between the two bistable states observed in our tailed-trench system (**Figure S5**).

References

1. Rittman, M., Gilroy, E., Koohy, H., Rodger, A. & Richards, A. Is dna a worm-like chain in couette flow?: In search of persistence length, a critical review. *Sci. progress* **92**, 163–204 (2009).
2. Ouldridge, T. E., Louis, A. A. & Doye, J. P. Structural, mechanical, and thermodynamic properties of a coarse-grained dna model. *The J. chemical physics* **134** (2011).
3. Sensale, S., Peng, Z. & Chang, H.-C. Kinetic theory for dna melting with vibrational entropy. *The J. Chem. Phys.* **147** (2017).
4. Naskar, S. & Maiti, P. K. Mechanical properties of dna and dna nanostructures: Comparison of atomistic, martini and oxdna models. *J. Mater. Chem. B* **9**, 5102–5113 (2021).
